# Construction of Engineered *Escherichia coli* and Optimization of Conditions for Carcinine Synthesis via Multi-Enzyme Cascade Catalysis

**DOI:** 10.3390/biom16081124

**Published:** 2026-08-01

**Authors:** Haoni Luan, Rui Yang, Wenhan Qiu, Kaiyue Feng, Wei Xu, Fei Wang, Wei Feng, Peng Song

**Affiliations:** 1 School of Pharmaceutical Sciences and Food Engineering, Liaocheng University, Liaocheng 252000, China; 2 Shandong Key Laboratory of Applied Technology for Protein and Peptide Drugs, Liaocheng 252000, China; 3 State Key Laboratory of Macromolecular Drugs and Large-Scale Preparation, Liaocheng 252000, China; 4 College of Agriculture and Biology, Liaocheng University, Liaocheng 252000, China

**Keywords:** carcinine, multi-enzyme cascade catalysis, fusion protein, CRISPR/Cas9, biosynthesis

## Abstract

Carcinine is an imidazole dipeptide with potent antioxidant and antiglycation properties, although its chemical synthesis currently relies on severely environmentally harmful processes. In this work, a multi-enzyme cascade biotransformation system comprising 4′-phosphopantetheinyl transferase and non-ribosomal peptide synthetase was constructed. To overcome the limitations arising from stochastic spatial distribution and suboptimal mass transfer associated with independent enzymes, a fusion protein strategy was adopted. The two enzymes were fused via a flexible genetic linker within plasmid pET28a-SFP-L-Ebony, which enabled robust soluble expression in *Escherichia coli*. Concurrently, the endogenous peptidase genes (*pepA*, *pepB*, *pepD*, and *pepN*) were systematically knocked out using CRISPR/Cas9-mediated gene editing. This quadruple protease-deficient strain (designated SFP-L-Ebony-Δ*pepABDN*) effectively suppressed product degradation. Subsequent optimization revealed that optimal catalytic performance occurred at 25 °C and pH 7.0. The highest biotransformation efficiency was achieved using 15 g/L crude enzymes, in the presence of 2 mM ATP and 10 mM MgCl_2_. Through a fed-batch substrate feeding strategy in a 50 mL reaction system, the final carcinine titer reached 7.0 g/L after 48 h. This study, therefore, provides an efficient and sustainable technological pathway for the green biomanufacturing of carcinine as well as other high-value dipeptides.

## 1. Introduction

**Figure 1:**
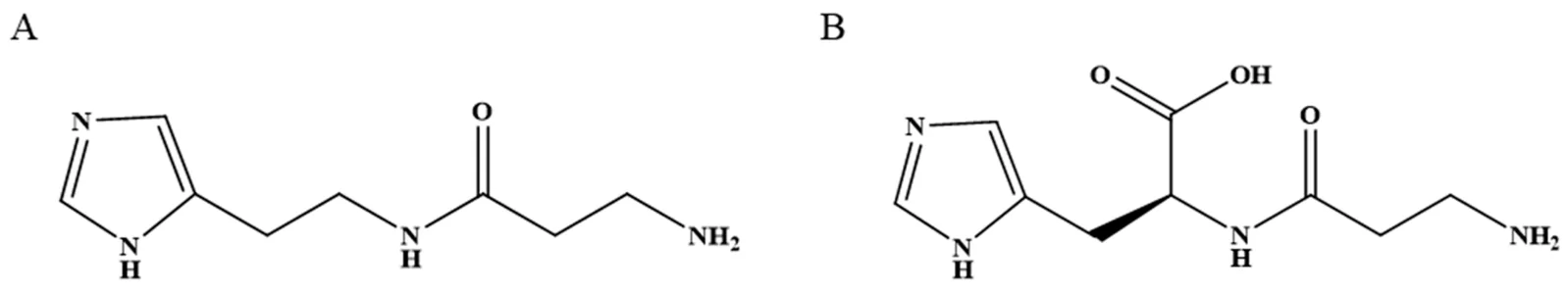
Chemical structures of carcinine and carnosine. (**A**) The structural formula of carcinine; (**B**) The structural formula of carnosine.

Carcinine, chemically designated as β-alanyl histamine, is an imidazole dipeptide comprising β-alanine and L-histamine (Figure 1). This compound was first isolated from crustaceans in 1975 and subsequently found in the mammalian heart [1]. Highly structurally analogous to carnosine, carcinine is a derivative formed through the reduction of the C-terminal carboxyl group of carnosine, thus retaining all of its active functional groups [2]. Consequently, carcinine exhibits biological activities that are consistent with those of carnosine. Notably, as carcinine is not recognized by carnosinase, it exhibits superior metabolic stability and a longer duration of action in the human body [3,4]. Furthermore, owing to the absence of a carboxyl group in its molecular structure, carcinine exhibits greater electropositivity and does not form internal salts [5]. This distinctive feature accounts for its higher adsorption capacity and greater permeability at the skin surface, as well as for its improved migration efficiency in biological tissues.

Owing to its metabolic stability and multifarious beneficial physiological functions, carcinine is considered a bioactive molecule with greater development potential than carnosine, and thus offers immense application prospects in the pharmaceutical, cosmetic, and functional food industries [6,7,8]. First, carcinine acts as a potent and stable antioxidant. It effectively scavenges ROS and RNS, and chelates pro-oxidant metal ions such as Cu^2+^, thereby protecting cell membranes, proteins, and DNA against oxidative damage [9,10]. Second, carcinine exhibits a remarkable anti-glycation capacity. By competitively binding to reducing sugars, it efficiently prevents the formation of advanced glycation end-products (AGEs). Given that AGE accumulation is intricately linked to the initiation and progression of skin aging, diabetic complications, and various degenerative diseases, carcinine holds substantial application value in anti-aging cosmetics and in the prevention and management of chronic diseases [11,12,13]. Furthermore, accumulating evidence indicates that carcinine may also possess other potential biological effects, including neuroprotection and pH buffering [14,15].

**Figure 2:**
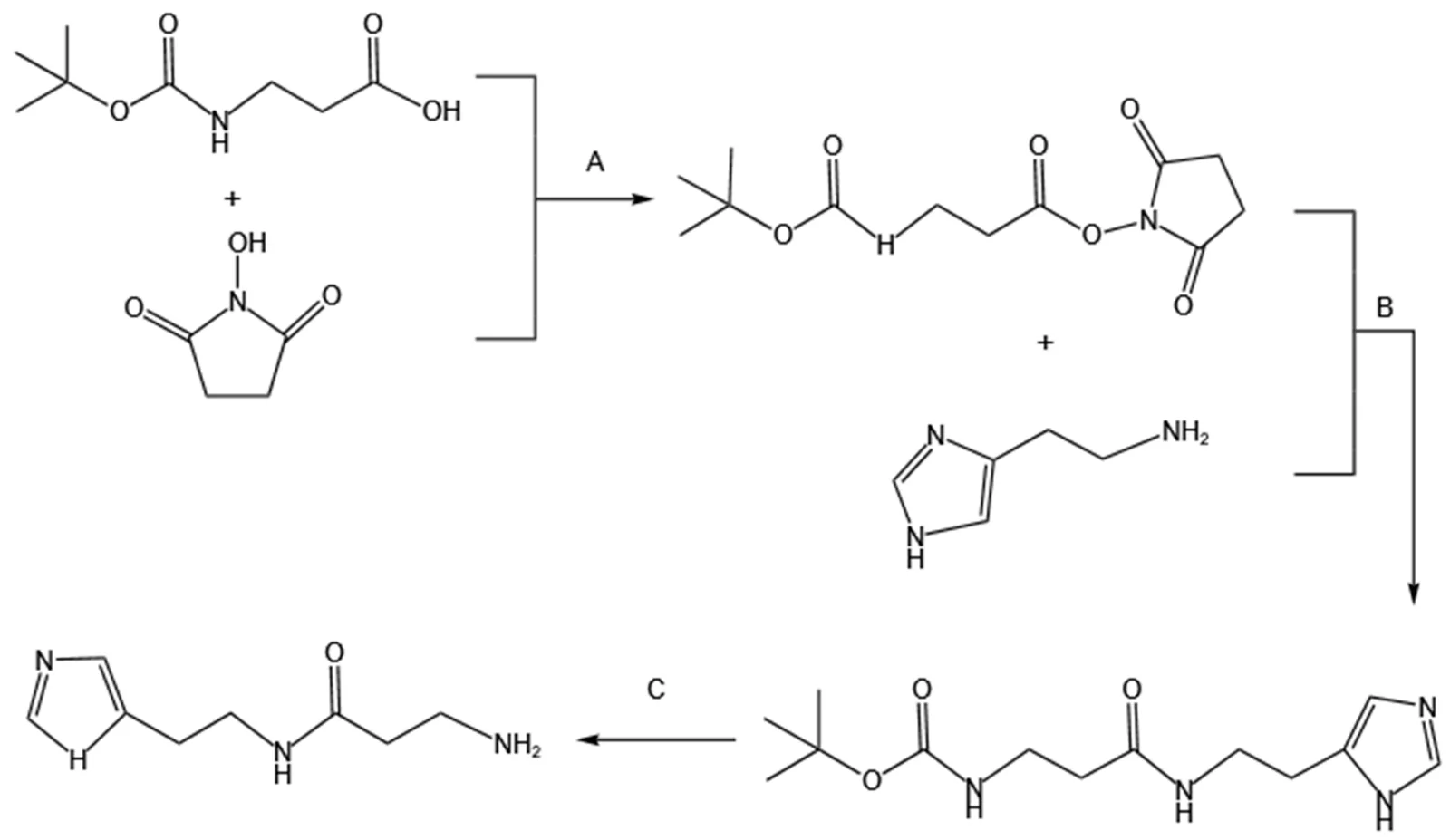
Chemical synthesis of carcinine. (**A**) Boc-β-Ala-OH reacts with N-Hydroxy succinimide to yield Boc-β-Ala-Osu; (**B**) Boc-β-Ala-OSu reacts with histamine to yield Boc-β-Ala-histamine; (**C**) Boc-β-Ala-histamine is deprotected to yield carcinine. Chemical structures were drawn using KingdrewPc_Edu_V4.0.0.20.

While multi-step chemical synthesis remains the predominant route for industrial carcinine production (Figure 2), it typically relies on protecting-group strategies (e.g., Boc-protection), chemical activating agents, and harsh cleavage reagents [16,17]. These multi-step chemical routes inevitably generate toxic by-products and corrosive acid effluents (e.g., trifluoroacetic acid or strong mineral acids during deprotection), presenting substantial downstream purification challenges and severe environmental compliance pressures [18,19,20,21,22]. Consequently, green enzymatic biotransformation has emerged as an attractive alternative, offering high regio- and stereoselectivity, mild aqueous reaction conditions, and superior environmental compatibility [23,24,25]. However, heterologous expression of the key non-ribosomal peptide synthetase (Ebony) in standard bacterial host strains remains severely bottlenecked by recalcitrant protein misfolding and low soluble expression yields [25,26,27].

Recent studies have established that β-alanine and histamine can be converted into carcinine via the NRPS-type enzyme Ebony, a process strictly dependent on post-translational phosphopantetheinylation by 4′-phosphopantetheinyl transferase (SFP) [23,28,29]. Although spatial linker engineering and whole-cell biocatalytic strategies have recently been explored to improve cascade catalytic efficiency [24,25], key synthetic bottlenecks persist. Most notably, heterologous expression of Ebony in *E. coli* is severely compromised by the formation of heavy inclusion bodies, which significantly limits the availability of a functional, active enzyme [25,26]. Furthermore, endogenous peptidases inherent to *E. coli* (e.g., *pepA*, *pepB*, *pepD*, and *pepN*) frequently degrade accumulated intracellular dipeptides, thereby diminishing overall product titers [30,31].

**Figure 3:**
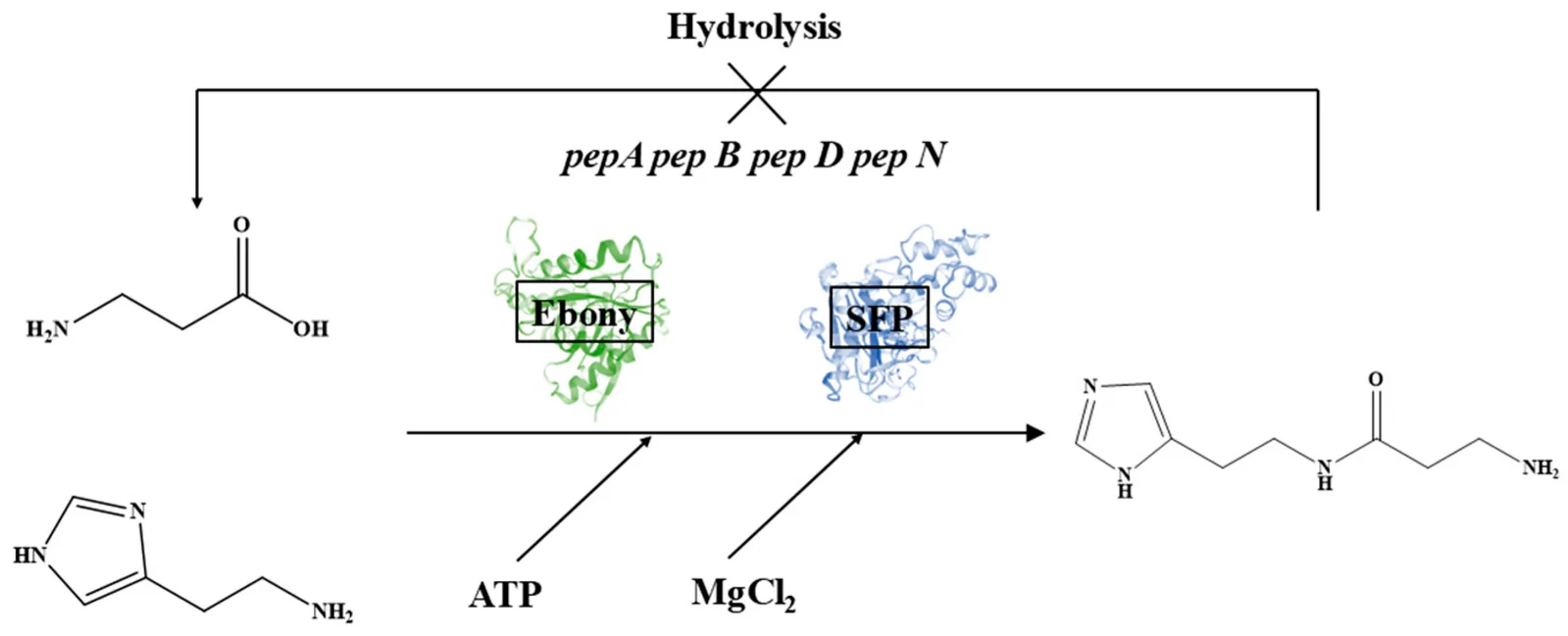
Schematic diagram of the biotransformational two-enzyme cascade system for carcinine synthesis. β-Alanine and histamine are converted into carcinine through the synergistic action of SFP and Ebony. In this system, ATP serves as a cosubstrate, and Mg^2+^ is an essential cofactor.

To overcome these critical limitations, this study reports a comprehensive genetic and enzyme engineering framework for high-efficiency carcinine biosynthesis. The overall biotransformation follows the stoichiometry: β-alanine + histamine + ATP → carcinine + AMP + PPi [32]. Candidate SFP homologs were first evaluated to identify the optimal activating enzyme, followed by the strategic incorporation of a C-terminal retro-protein XXA solubility tag to resolve the insoluble expression barrier of Ebony. Furthermore, spatial dual-enzyme tethering was achieved via a flexible genetic linker, while a CRISPR/Cas9-mediated quadruple peptidase gene deletion (Δ*pepABDN*) was implemented to suppress product degradation (Figure 3). This integrated biocatalytic platform establishes a robust and sustainable strategy for the green biomanufacturing of carcinine and related high-value dipeptide derivatives.

## 2. Materials and Methods

### 2.1. Materials and Chemicals

β-Alanine, histamine, adenosine triphosphate (ATP), and carcinine dihydrochloride (purity ≥98%, molecular weight: 255.1 g/mol) were obtained from Macklin Biochemical Co., Ltd. (Shanghai, China). DNA polymerases and restriction endonucleases (e.g., *Nco I*, *Nde I*, *Bsa I,* and *BamH I*) were purchased from Thermo Fisher Scientific (Waltham, MA, USA). Plasmid miniprep kits and DNA gel purification kits were obtained from Omega Bio-tek (Norcross, GA, USA). Unless otherwise specified, all other chemicals and reagents used in this study were of analytical grade and were obtained from commercial sources.

### 2.2. Strains and Plasmids

*E. coli* BL21(DE3) [genotype: *F– omp*T *hsdS_B_* (r_B_–, m_B_–) *gal dcm lon* λ(DE3)] and its engineered derivatives were used as host cells for carcinine biosynthesis. Gene expression was carried out using the pET28a(+) vector. The target genes encoding Ebony, SFP-1 from *Pseudomonas putida* (GenBank accession no. AAN66807), SFP-2 from *Bacillus subtilis* (GenBank accession no. X63158), and SFP-3 from *Pseudomonas aeruginosa* (GenBank accession no. AAG04554) were codon-optimized and synthesized by GenScript Biotech Co., Ltd. (Nanjing, China) [33,34,35,36]. Detailed information on the plasmids and strains used in this study is provided in Table S1. To construct a protease-deficient strain, four endogenous genes (*pepA*, *pepB*, *pepD*, and *pepN*) were sequentially knocked out in *E. coli* BL21(DE3) using the CRISPR/Cas9 system. The primers used for chromosomal gene deletion are listed in Table S2. Transformants were verified by colony PCR (Figure S1).

### 2.3. Gene Cloning

The four target genes (*ebony*, *sfp-1*, *sfp-2*, and *sfp-3*) were successfully cloned into the pET28a(+) vector and subsequently transformed into *E. coli* BL21(DE3), yielding the recombinant strains BL21-Ebony, BL21-SFP-1, BL21-SFP-2, and BL21-SFP-3, respectively.

### 2.4. Protein Overexpression and SDS-PAGE Analysis

Single colonies of the recombinant strains (designated BL21-Ebony, BL21-SFP-1, BL21-SFP-2, and BL21-SFP-3) were picked from Luria–Bertani (LB) agar plates and inoculated into liquid LB medium (5 g/L yeast extract, 10 g/L peptone, and 10 g/L NaCl) supplemented with 30 μg/mL kanamycin, then cultured at 37 °C with shaking at 220 rpm. When the OD_600_ reached 0.4–0.6, the pre-cultures were transferred at 2% (*v*/*v*) into Terrific Broth (TB) medium (24 g/L yeast extract, 12 g/L peptone, 4 g/L glycerol, 16.4 g/L K_2_HPO_4_, and 2.32 g/L KH_2_PO_4_). The TB cultures were incubated under the same conditions (37 °C, 220 rpm with shaking) until the optical density at 600 nm (OD_600_) reached 1.0. Gene overexpression was then induced by adding 0.3 mM isopropyl-β-D-thiogalactopyranoside (IPTG), followed by further incubation at 25 °C with shaking at 220 rpm for 16 h.

The cultured cells were transferred to centrifuge tubes and harvested by centrifugation at 8000× *g* and 4 °C for 10 min, after which the supernatant was removed. The resulting cell pellets were washed by resuspension in 10 mL of Tris–HCl buffer (50 mM, pH 7.5), centrifuged again at 4 °C for 8 min, and the supernatant was discarded. The washed cell pellets were then weighed, resuspended in Tris–HCl buffer to a final concentration of 0.1 g/mL, and subsequently lysed by a high-pressure homogenizer at 1200 bar. To remove cell debris, the lysates were subjected to centrifugation at 8000× *g* and 4 °C for 10 min. The resulting supernatant was mixed with SDS-PAGE loading buffer and heated at 100 °C for 5 min. Finally, 5 μL of each protein sample was loaded onto a 12% sodium dodecyl sulfate–polyacrylamide gel to analyze the expression of the recombinant proteins.

### 2.5. Optimization of Expression Conditions for Recombinant E. coli BL21-Ebony and BL21-SFP

#### 2.5.1. Optimization of Induction Conditions

Induction temperature and IPTG concentration are critical parameters that govern heterologous protein expression. To address inclusion body formation, we investigated whether lowering the induction temperature and reducing the IPTG concentration for the Ebony enzyme could slow cell growth, limit over-expression of the foreign protein, and consequently facilitate proper protein folding. Furthermore, the effects of different induction temperatures (20–30°C) and IPTG concentrations (0.1–1.0 mM) on the expression level and catalytic activity of the SFP enzyme were examined. A one-factor-at-a-time experimental design was adopted, in which each parameter was varied sequentially while the others were kept constant, thus allowing determination of the specific impact of each variable on enzyme expression and catalytic activity.

#### 2.5.2. Fusion of Solubility-Enhancing Tags

To improve the soluble expression of the Ebony enzyme, retro-protein XXA tags were introduced at either its C-terminus or N-terminus [26,37]. The tag-fused sequences were synthesized by GenScript Biotech Co., Ltd. (Nanjing, China). These sequences were then cloned into pET28a-Ebony to yield the recombinant expression vectors pET28a-Ebony-C and pET28a-Ebony-N, which were subsequently transformed into *E. coli* BL21(DE3) to generate the recombinant strains BL21-Ebony-C and BL21-Ebony-N, respectively. Protein induction was carried out at 30 °C, followed by assessment of protein expression levels and enzymatic activity assays.

### 2.6. Biotransformation and Analytical Methods

For the biotransformation assays, recombinant cells expressing Ebony-C, Ebony-N, SFP-1, SFP-2, and SFP-3 (induced with IPTG for 16 h) were harvested by centrifugation at 8000× *g* and 4 °C for 10 min. The cell pellets were resuspended in 50 mM Tris–HCl buffer (pH 7.5) and lysed by a high-pressure homogenizer. Biotransformation reactions were subsequently performed by combining each of the two Ebony constructs with each of the three SFP variants in a pairwise manner. The reaction mixture was prepared in 50 mM Tris–HCl buffer (pH 7.0) and contained 10 g/L crude enzymes (wet cell weight equivalent), 2 mM ATP, 10 mM MgCl_2_, 10 mM β-alanine, and 20 mM histamine; incubation was carried out at 25 °C [23]. At 12 h intervals, 100 μL aliquots were withdrawn and immediately mixed with 900 μL of acetonitrile to quench the reaction. All experiments were performed in triplicate.

The concentration of carcinine in the reaction mixture was determined by derivatization with DNS-Cl, followed by HPLC analysis. After quenching the reaction with acetonitrile, the samples were centrifuged at 12,000 rpm for 10 min. A 200 μL aliquot of the resulting supernatant was transferred to a 1.5 mL centrifuge tube and mixed with 20 μL of NaOH (2 M), 60 μL of saturated NaHCO_3_ solution, and 400 μL of DNS-Cl (5 mg/mL in acetone). The mixture was incubated at 37 °C for 30 min, and the derivatization reaction was subsequently terminated by adding 25 μL of aqueous ammonia [24]. Before HPLC analysis, the samples were filtered through a 0.22-μm nylon membrane filter. Quantification was performed on a Yilite UV3100 series HPLC system (Dalian Yilite Analytical Instruments Co., Ltd., Dalian, China) equipped with a Yilite C18 column (5 μm, 4.6 mm × 250 mm). The mobile phase was 70% acetonitrile and 30% water (*v*/*v*), with a flow rate of 1 mL/min. The detection wavelength was set at 245 nm, and the injection volume was 10 μL. Under these conditions, the retention time of derivatized carcinine was approximately 8 min (Figure S2). Carcinine quantification was performed using a standard curve prepared with carcinine dihydrochloride (molecular weight: 255.1 g/mol) as the reference standard. The calibration curve was constructed using serial dilutions of the standard over a concentration range of 0.125–2.0 g/L and exhibited excellent linearity (R^2^ > 0.999). To maintain consistency with the free-base form commonly used in the literature, all reported titers were converted to carcinine free base equivalents (molecular weight: 182.2 g/mol) using the conversion factor 182.2/255.1 = 0.714.

### 2.7. Construction of the Single-Strain Dual-Enzyme Co-Expression System

To achieve highly efficient spatial synergy between the activating enzyme SFP-3 and the synthetase Ebony, a fusion protein strategy was employed in which a flexible linker peptide sequence (GGGGSGGGS) was introduced between the two enzymes to generate the fusion co-expression strain [25]. The target gene sequence sfp-L-ebony was codon-optimized and synthesized by GenScript Biotech Co., Ltd. (Nanjing, China). The synthesized fragment was then cloned into the linearized pET28a(+) expression vector by double digestion and ligation to yield the recombinant fusion expression plasmid pET28a-SFP-L-Ebony. For transformation, 10 μL of the ligation product was mixed with 50 μL of *E. coli* DH5α competent cells. The mixture was incubated on ice for 20 min, then heat-shocked at 42 °C for 90 s, and immediately returned to ice for 2 min. Subsequently, 1 mL of LB medium was added, and the cells were incubated at 37 °C with shaking at 200 rpm for 50 min for phenotypic recovery. The culture was then centrifuged, the supernatant was partially removed, and the cell pellet was resuspended in the remaining medium before plating on selective LB agar plates containing 50 μg/mL kanamycin. After sequence verification, the correct fusion plasmid was transformed into competent *E. coli* BL21(DE3) cells by heat shock. Transformants were screened and selected on LB agar plates containing kanamycin (50 μg/mL) to obtain the engineered strain BL21(DE3)-SFP-L-Ebony. Protein expression after induction and subsequent biotransformation assays for this strain were assessed by SDS-PAGE and conducted following the procedures described in Section 2.4 and Section 2.6, respectively.

### 2.8. Construction of the Protease-Deficient Strains

To construct the protease-deficient strains, we employed the pEcCas/pEcgRNA system described by Li et al. [38]. Gene deletions were performed sequentially in the following order: *pepB*, *pepD*, *pepA*, and *pepN*. For each target gene, sgRNA oligos (Table S2) were annealed and cloned into the *BsaI*-linearized pEcgRNA vector. Donor DNA fragments serving as homologous repair templates were generated by amplifying approximately 500 bp regions upstream and downstream of each target gene. The sgRNA-expressing plasmids and their corresponding donor DNA fragments were co-electroporated into competent cells harboring the pEcCas plasmid. After each round of deletion, positive recombinants were screened by colony PCR using validation primers located outside the deletion regions (Table S2), and the PCR products were analyzed by gel electrophoresis (Figure S1). Plasmid curing was subsequently achieved by rhamnose-induced loss of pEcgRNA, followed by sucrose-based counter-selection of pEcCas, resulting in the quadruple-peptidase-deficient strain BL21(DE3)-Δ*pepABDN*. Finally, the fusion expression plasmid pET28a-SFP-L-Ebony was transformed into this protease-deficient host by heat shock, and transformants were selected on LB agar plates containing 50 μg/mL kanamycin to generate the engineered strain SFP-L-Ebony-Δ*pepABDN*. SDS-PAGE analysis and multi-enzyme cascade catalytic reactions for this strain were performed following the methods described in Section 2.4 and Section 2.6, respectively.

### 2.9. Characterization of Enzymatic Properties

#### 2.9.1. Effect of Temperature on Enzymatic Activity and Stability

The optimum reaction temperature for the fusion enzyme SFP-L-Ebony-Δ*pepABDN* was evaluated across a temperature range of 15–35 °C in a standard assay mixture (50 mM Tris-HCl buffer, pH 7.0). Relative enzymatic activity was determined by normalizing the measured values against the maximum activity observed at the optimum temperature (set as 100%). To evaluate the thermal stability of the fusion enzyme, it was pre-incubated in 50 mM Tris-HCl buffer (pH 7.0) at 30 °C and 50 °C in a water bath. Aliquots were withdrawn at specified intervals (0, 4, 8, 12, 16, and 20 h) and immediately cooled in an ice bath. Residual activity was subsequently measured under standard assay conditions. The activity of the untreated enzyme was set as 100% for calculating thermal stability.

#### 2.9.2. Effect of pH on Enzymatic Activity and Stability

The optimum pH for the fusion enzyme SFP-L-Ebony-Δ*pepABDN* was determined at 25 °C over a pH range of 6.0–9.0 in 50 mM Tris-HCl buffer. Relative activity was calculated by normalizing the measured values to the maximum activity observed at the optimum pH (set as 100%). To assess pH stability, the fusion enzyme was pre-incubated in buffers at pH 5.0 and pH 10.0 for specified time intervals. Residual enzymatic activity was subsequently measured under standard conditions, with the activity of the untreated enzyme set as 100%.

### 2.10. Optimization of Biotransformation Conditions

To optimize the reaction conditions for the multi-enzyme cascade catalytic system, a systematic investigation was conducted in a 10-mL reaction volume to evaluate the effects of critical parameters, namely SFP-L-Ebony crude enzyme concentration (5–30 g/L), reaction time (6–60 h), pH (6.0–8.0), temperature (15–35 °C), ATP concentration (2–10 mM), and MgCl_2_ concentration (5–25 mM). A one-factor-at-a-time experimental design was adopted, in which each parameter was varied sequentially while the others were kept constant, thus allowing determination of the specific impact of each variable on biotransformation efficiency.

### 2.11. Scale-Up Biotransformation

To evaluate the scale-up potential of the biotransformation process, the reaction was carried out in an incubator equipped with a magnetic stirrer and maintained in a constant-temperature water bath at 25 °C. The initial reaction mixture contained crude enzymes prepared from the engineered strain SFP-L-Ebony-Δ*pepABDN* at a concentration of 15 g/L, 20 mM β-alanine, 40 mM histamine, 10 mM MgCl_2_, and 2 mM ATP, in 50 mM Tris–HCl buffer (pH 7.0). A fed-batch strategy was implemented by adding 20 mM β-alanine and 2 mM ATP after 24 h of reaction. Aliquots were withdrawn at 12-h intervals and subsequently analyzed by HPLC under the same conditions described in Section 2.6.

## 3. Results

### 3.1. Heterologous Expression of Recombinant Strains

**Figure 4:**
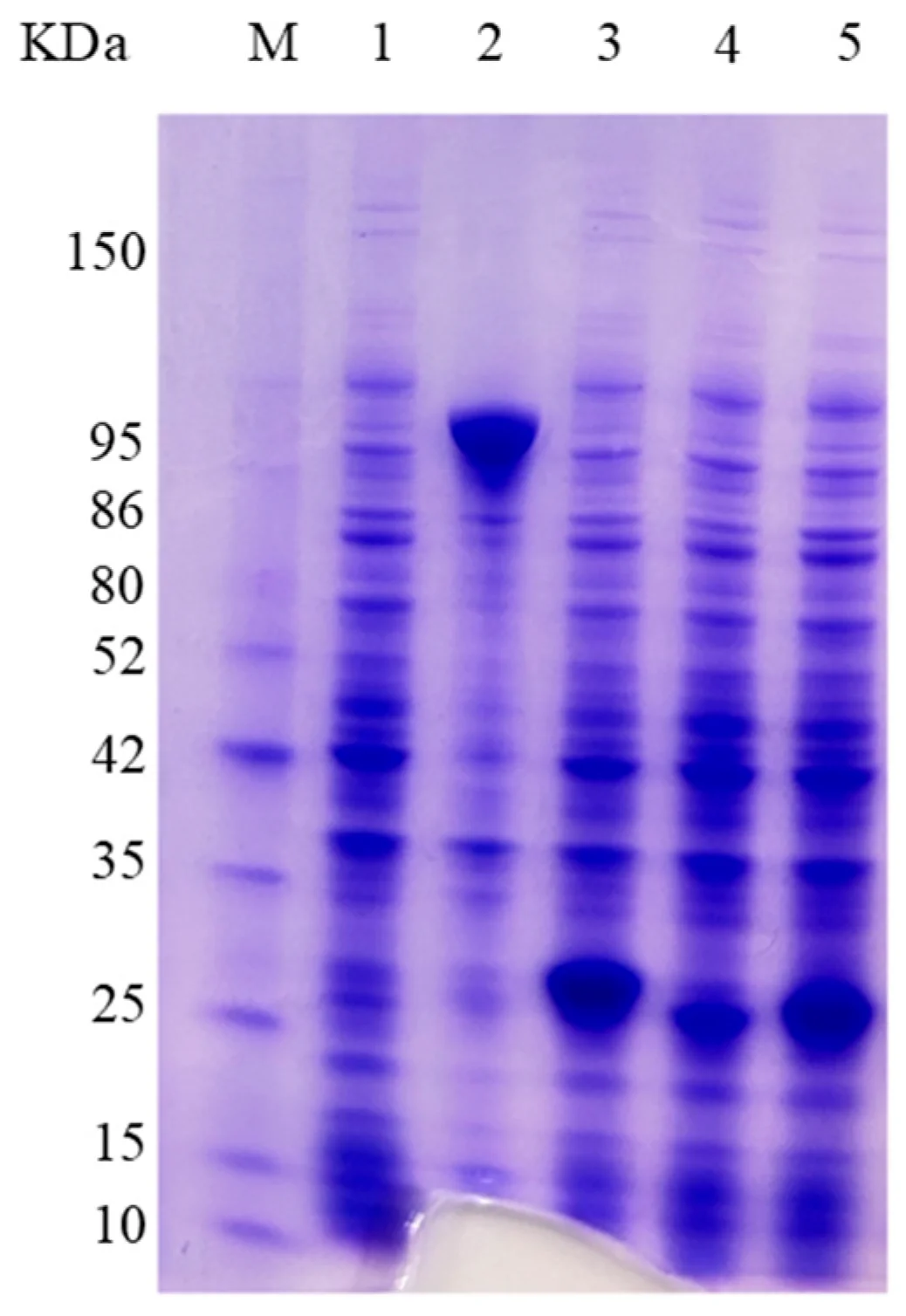
SDS-PAGE analysis of recombinant Ebony and SFP variants expressed in *E. coli* BL21(DE3). lane 1: culture supernatants from recombinant strains BL21(DE3)-Ebony; lane 2: culture pellets from recombinant strains BL21(DE3)-Ebony; lane 3: culture supernatants from recombinant strains BL21(DE3)-SFP-1; lane 4: culture supernatants from recombinant strains BL21(DE3)-SFP-2; lane 5: culture supernatants from recombinant strains BL21(DE3)-SFP-3. The original Western blot images can be found in the Supplementary Materials.

The theoretical molecular masses of the Ebony and the SFP are approximately 98 kDa and 26 kDa, respectively. To evaluate their expression levels, the recombinant *E. coli* BL21(DE3) strains were induced with 0.3 mM IPTG at 25 °C for 16 h. As illustrated in Figure 4, all three investigated SFPs were successfully expressed in their soluble forms. In contrast, the expression of the Ebony resulted in the formation of inclusion bodies, appearing predominantly in the insoluble fraction.

### 3.2. Optimization and Analysis of Induction Conditions

**Figure 5:**
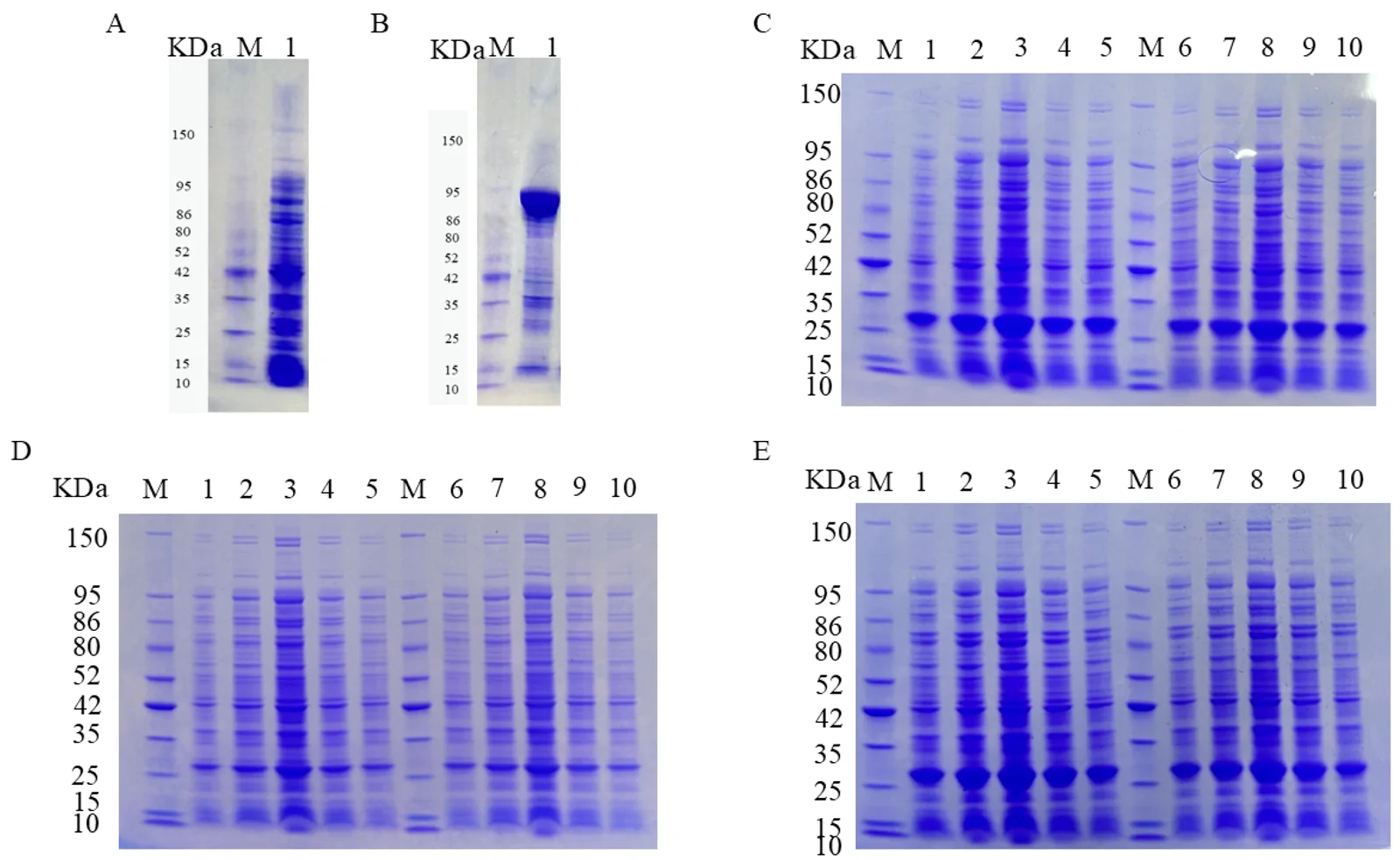
SDS-PAGE analysis of Ebony and SFP variants under different induction conditions. (**A**) Lane 1: low-temperature culture supernatants from recombinant strains BL21(DE3)-Ebony. (**B**) Lane 1: low-temperature culture pellets from recombinant strains BL21(DE3)-Ebony. (**C**) Optimization of induction temperature and inducer concentration for the recombinant strain BL21(DE3)-SFP-1. Lane 1: induced at 20 °C. Lane 2: induced at 23 °C. Lane 3: induced at 25 °C. Lane 4: induced at 28 °C. Lane 5: induced at 30 °C. Lane 6: induction with 0.1 mM IPTG. Lane 7: induction with 0.3 mM IPTG. Lane 8: induction with 0.5 mM IPTG. Lane 9: induction with 0.8 mM IPTG. Lane 10: induction with 1 mM IPTG. (**D**) Optimization of induction temperature and inducer concentration for the recombinant strain BL21(DE3)-SFP-2. (**E**) Optimization of induction temperature and inducer concentration for the recombinant strain BL21(DE3)-SFP-3. The original Western blot images can be found in the Supplementary Materials.

Despite systematically lowering the induction temperature and reducing the IPTG concentration for the Ebony enzyme, we observed no discernible differences in its protein expression profiles. The target enzyme remained exclusively localized in the insoluble pellet, persisting as inclusion bodies (Figure 5A,B). Conversely, the SFP enzymes exhibited their highest expression levels when induced at 25 °C with 0.5 mM IPTG (Figure 5C–E). This behavioral discrepancy can be attributed to the delicate balance between cellular metabolism and protein folding dynamics. Hypothermic conditions inevitably reduce the specific growth rate of the host strain, thus suppressing the overall protein synthesis rate. Conversely, elevated induction temperatures accelerate translation, which significantly increases the propensity for protein misfolding [39]. Regarding the inducer, insufficient IPTG concentrations lead to inadequate transcriptional activation, whereas excessive concentrations may exert notable cytotoxicity or induce overwhelming protein aggregation and misfolding.

### 3.3. Soluble Expression of Tag-Fused Ebony

**Figure 6:**
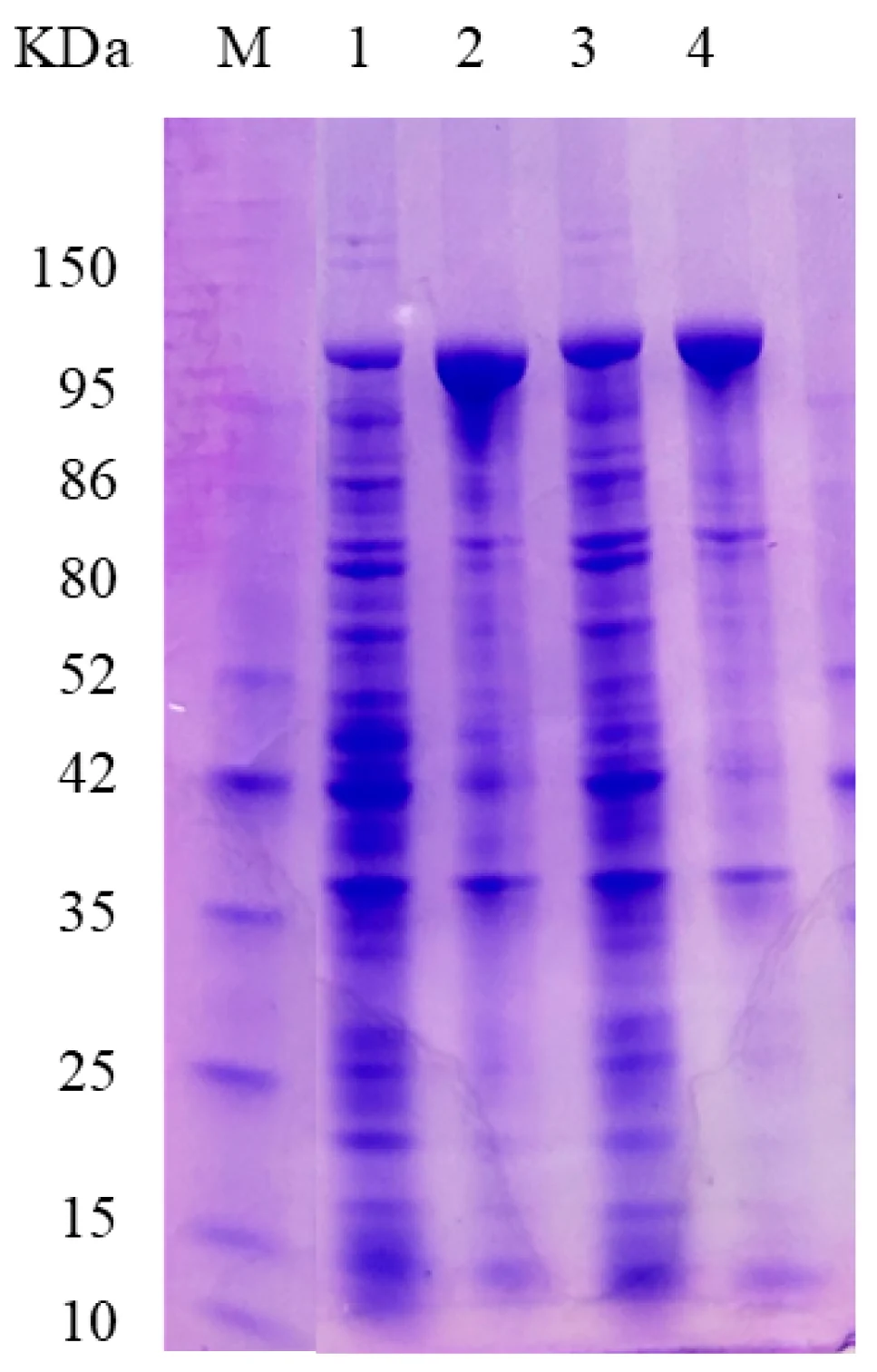
SDS-PAGE analysis of soluble expression of Ebony-C and Ebony-N fusion proteins with the XXA tag. Lane 1: culture supernatants from recombinant strains BL21(DE3)-Ebony-C; lane 2: culture pellets from recombinant strains BL21(DE3)-Ebony-C; lane 3: culture supernatants from recombinant strains BL21(DE3)-Ebony-N; lane 4: culture pellets from recombinant strains BL21(DE3)-Ebony-N. The original Western blot images can be found in the Supplementary Materials.

Following fusion of the retro-protein XXA tag to either the C-terminus or N-terminus of the target protein, the theoretical molecular weight of the engineered Ebony-XXA construct increased to approximately 117 kDa. After inducing the recombinant *E. coli* strains with 0.3 mM IPTG at 30 °C for 16 h, the non-ribosomal peptide synthetase fusion was successfully expressed in soluble form (Figure 6). This result demonstrates that the introduction of the retro-protein XXA tag can effectively circumvent inclusion body formation even under relatively high induction temperatures (30 °C), thus significantly facilitating downstream purification and offering substantial advantages for potential industrial-scale production.

### 3.4. Analysis of Biotransformation Capacity of Recombinant Strains

**Figure 7:**
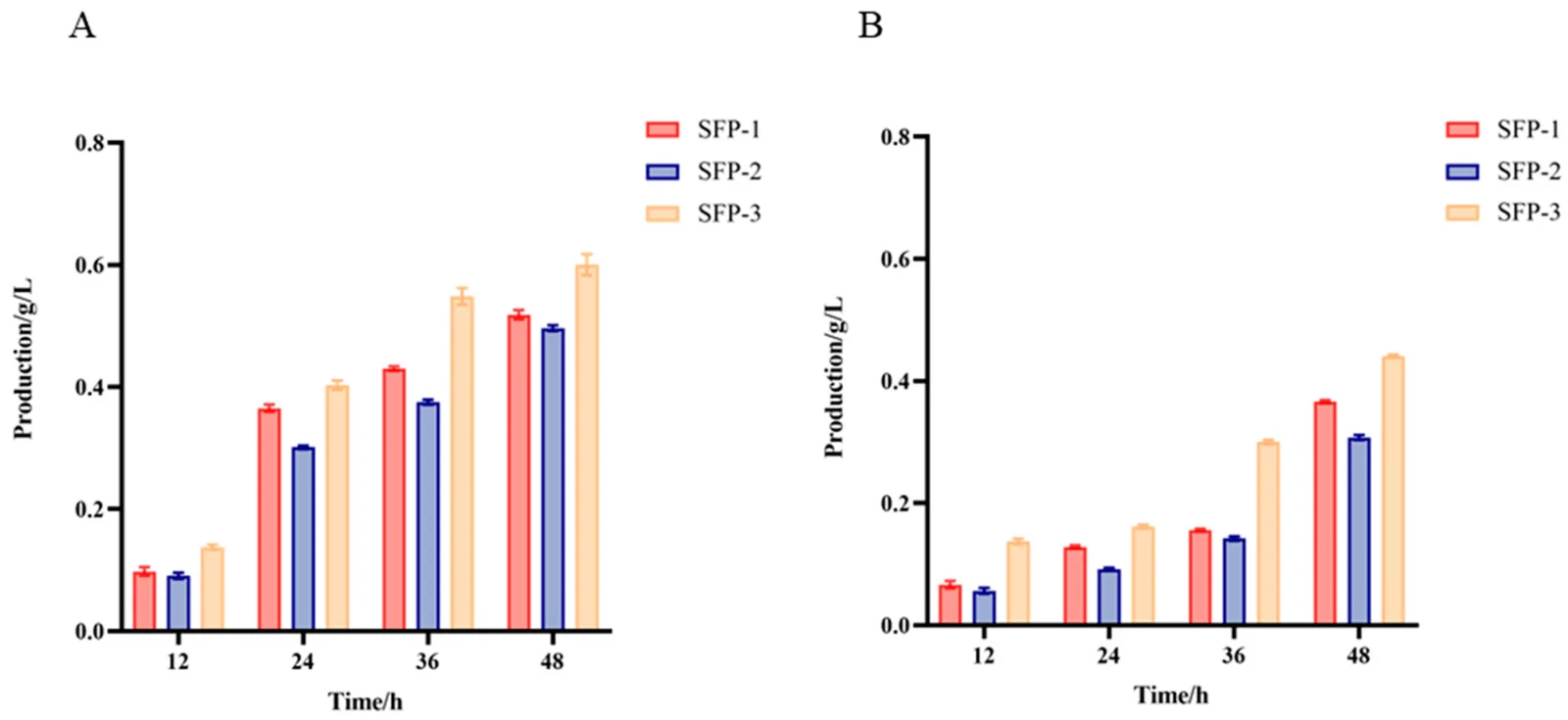
Synthesis of carcinine using different catalytic systems. (**A**) Synthesis of carcinine via reaction of Ebony-C with SFP-1, SFP-2 and SFP-3, respectively. (**B**) Synthesis of carcinine via reaction of Ebony-N with SFP-1, SFP-2 and SFP-3, respectively. Data are presented as mean ± SD, *n* = 3.

To evaluate the carcinine synthesis efficiency of the Ebony constructs (Ebony-C and Ebony-N) when coupled with different SFP variants, a 48 h biotransformation reaction was performed using the various multi-enzyme catalytic systems. The results showed that all investigated enzyme combinations exhibited catalytic activity, albeit with varying efficiencies (Figure 7). Notably, the catalytic system comprising Ebony-C and SFP-3 displayed superior catalytic performance, yielding significantly higher carcinine production across all monitored time intervals than the other combinations. Consequently, the BL21(DE3)-Ebony-C and BL21(DE3)-SFP-3 systems were selected for all subsequent experiments.

### 3.5. Expression and Functional Analysis of the Single-Strain Dual-Enzyme Co-Expression System

**Figure 8:**
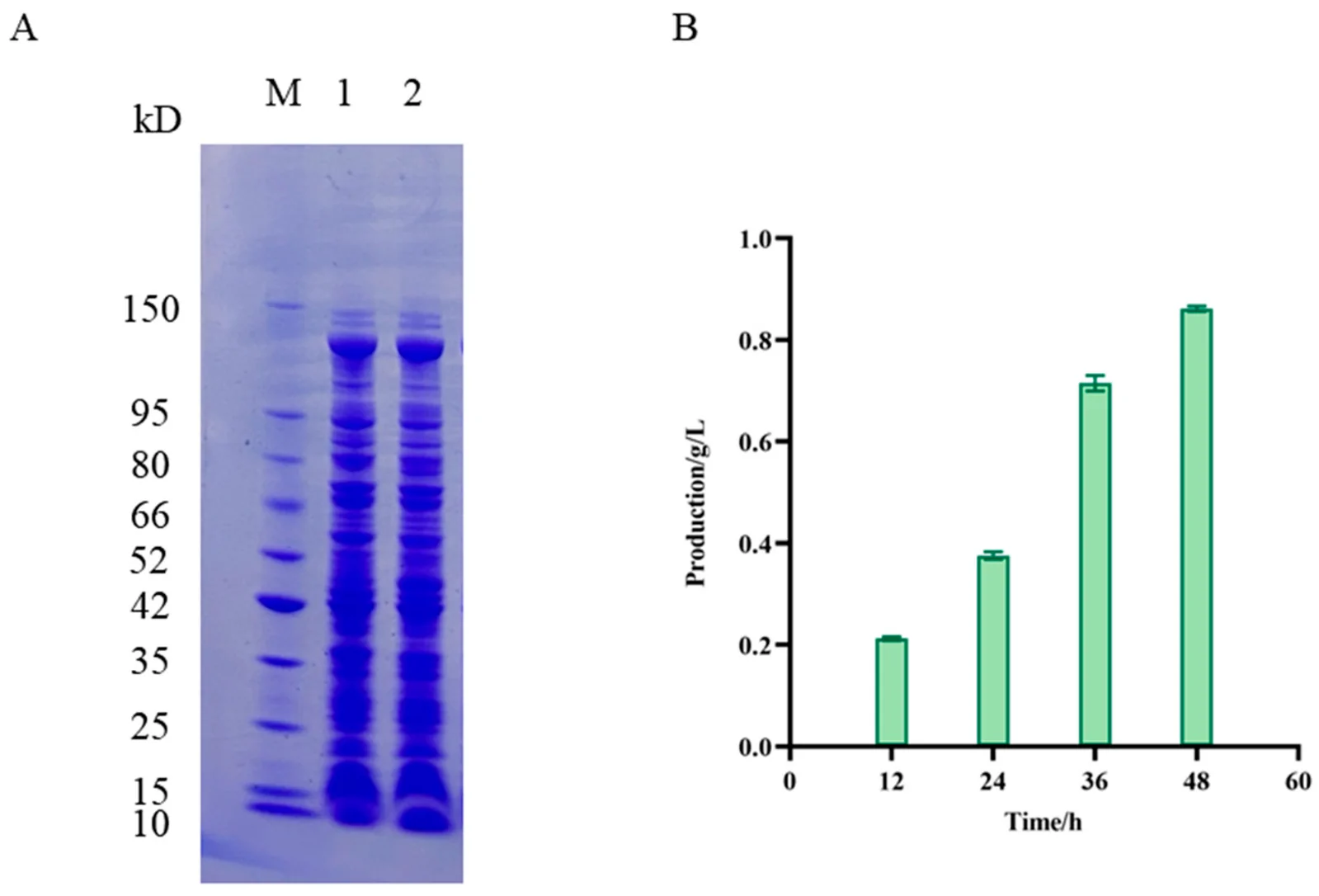
(**A**) SDS-PAGE analysis of the fusion protein SFP-L-Ebony expressed in BL21(DE3) and the protease-deficient strain SFP-L-Ebony-Δ*pepABDN*. Lane 1: culture supernatants from recombinant strains BL21(DE3)-SFP-L-Ebony. Lane 2: culture pellets from recombinant strains SFP-L-Ebony-Δ*pepABDN*. (**B**) Synthesis of carcinine via reaction of BL21(DE3)-SFP-L-Ebony. Data are presented as mean ± SD, *n* = 3. The original Western blot images can be found in the Supplementary Materials.

To shorten the spatial distance between the heterologous activating enzyme and the upstream synthetase, and thereby promote the in situ post-translational modification of Ebony, a fusion protein strategy was employed to construct the recombinant plasmid pET28a-SFP-L-Ebony. This recombinant plasmid was subsequently transformed into *E. coli* BL21(DE3) for protein expression upon induction. Following cell lysis, the expression products were analyzed by SDS-PAGE. As shown in Figure 8A, a distinct target band appeared at approximately 143 kDa, which was highly consistent with the theoretical molecular weight of the SFP-L-Ebony fusion protein. Furthermore, biotransformation assays were conducted using crude enzymes with β-alanine and histamine as substrates. The results showed the distinct superiority of the BL21(DE3)-SFP-L-Ebony strain in carcinine biotransformation, achieving a 1.44-fold increase (44% enhancement) in carcinine yield over the single-strain, single-enzyme catalytic system (Figure 8B). This notable improvement suggests that flexible concatenation of the activating enzyme SFP-3 and the synthetase Ebony via the genetic linker effectively achieved spatial “functional tethering”, thereby enhancing metabolic flux via enhanced enzyme proximity [40].

### 3.6. Expression of the Fusion Protein in the Protease-Deficient Strain

To enhance the metabolic stability of the target product, a CRISPR/Cas9 genome editing system was employed to systematically delete four major endogenous peptidase-encoding genes (*pepA*, *pepB*, *pepD*, and *pepN*) from the chromosome of *E. coli* BL21(DE3). The successful construction of this quadruple-peptidase-deficient strain was verified by genomic sequencing, and the resulting host was designated BL21(DE3) Δ*pepABDN*. Subsequently, the fusion expression vector pET28a-SFP-L-Ebony was transformed into this protease-deficient host to generate the engineered strain SFP-L-Ebony-Δ*pepABDN*. Following induction with 0.3 mM IPTG at 25 °C for 16 h, the whole-cell lysates were analyzed by SDS-PAGE, which confirmed successful soluble expression of the target fusion protein (Figure 8A). Notably, the quadruple peptidase deficiency did not affect fusion protein expression, confirming that the genetic modifications impose no significant metabolic burden.

### 3.7. Enzymatic Properties of SFP-L-Ebony-ΔpepABDN

#### 3.7.1. Optimum Temperature and Thermal Stability

**Figure 9:**
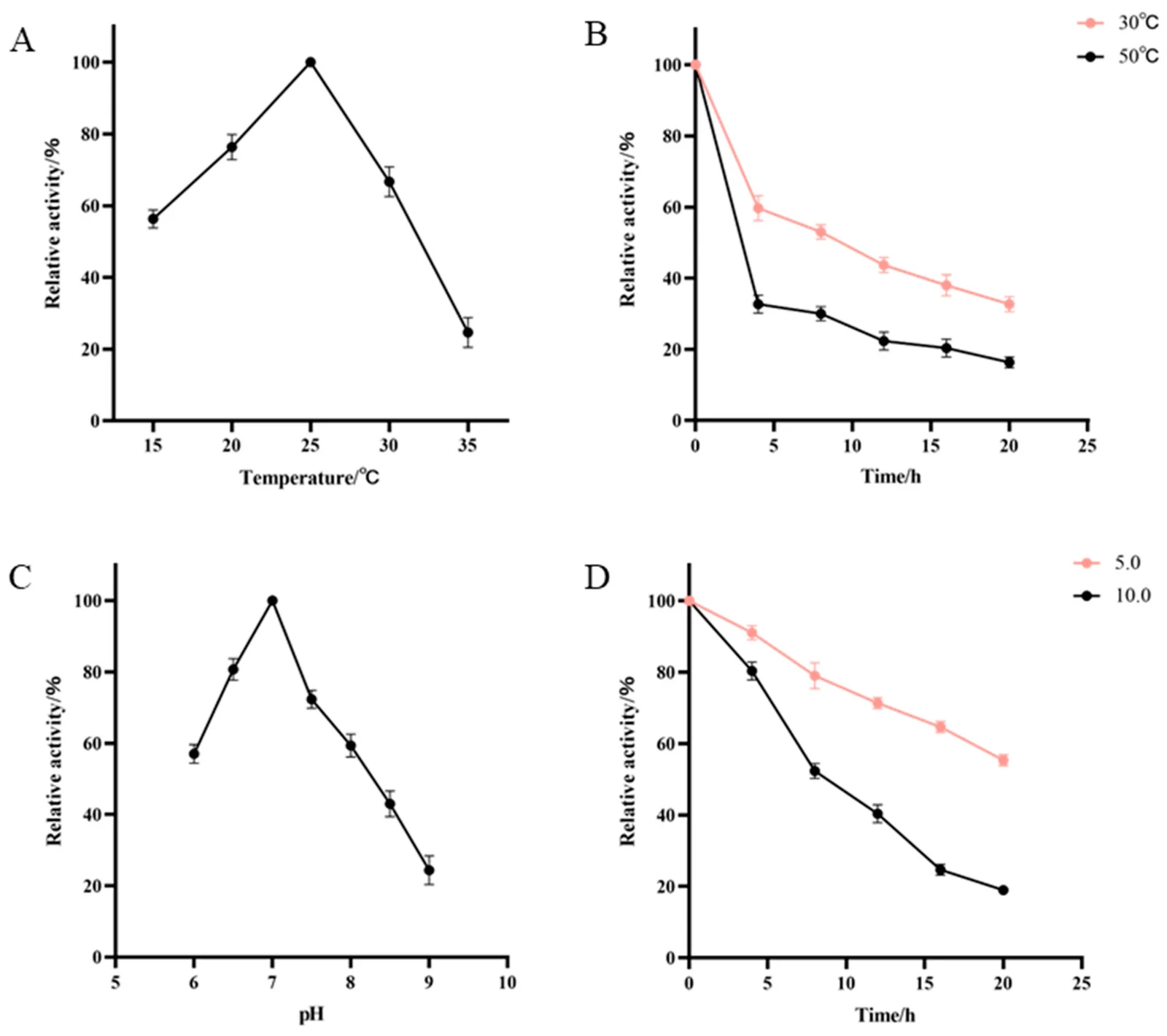
Effects of pH and different temperatures on SFP-L-Ebony-Δ*pepABDN* activity. (**A**) Optimum temperature of SFP-L-Ebony-Δ*pepABDN*. (**B**) Temperature stability of SFP-L-Ebony-Δ*pepABDN*. The enzyme was incubated at 30 °C and 50 °C for up to 20 h, and residual activities were measured at the indicated time points. Half-lives (t_1/2_) calculated from first-order decay fitting were 12.3 ± 0.5 h at 30 °C and 7.3 ± 0.4 h at 50 °C. (**C**) Optimum pH of SFP-L-Ebony-Δ*pepABDN*. (**D**) pH stability of SFP-L-Ebony-Δ*pepABDN*. The enzyme was pre-incubated at pH 5.0 and pH 10.0 for up to 20 h, and residual activities were measured at the indicated time points. Half-lives (t_1/2_) calculated from first-order decay fitting were 24.2 ± 0.6 h at pH 5.0 and 8.3 ± 0.2 h at pH 10.0. Data are presented as mean ± SD, *n* = 3.

To investigate the temperature dependence of the fusion enzyme SFP-L-Ebony-Δ*pepABDN* activity, enzymatic assays were performed across a temperature range of 15–35 °C at pH 7.0. The results showed that the enzyme exhibited its maximum activity at 25 °C. Within the temperature range of 15–25 °C, the relative activity of the fusion enzyme increased progressively with rising temperature; however, a sharp decline in activity was observed when the temperature reached 30 °C or above (Figure 9A). To evaluate the thermal stability of the fusion enzyme, it was incubated in a metal block bath at 30 °C and 50 °C, with its residual activity monitored at regular intervals. As shown in Figure 9B, the fusion enzyme retained approximately 32% of its initial activity after incubation at 30 °C for 20 h, whereas it retained 16% of its initial activity following incubation at 50 °C. First-order decay fitting of the inactivation curves yielded half-lives (t_1/2_) of 12.3 ± 0.5 h at 30 °C and 7.3 ± 0.4 h at 50 °C, with corresponding inactivation rate constants (k) of 0.0564 ± 0.0023 h^−1^ and 0.0950 ± 0.0054 h^−1^, respectively (Figure 9B).

#### 3.7.2. Optimum pH and pH Stability

The enzymatic activity of the fusion enzyme SFP-L-Ebony-Δ*pepABDN* was measured at 25 °C in buffers across a pH range of 6.0–9.0. The enzyme exhibited its optimal activity at pH 7.0. Between pH 6.0 and 7.0, the relative enzymatic activity increased gradually with increasing pH, whereas a steady decline was observed from pH 7.0 to 9.0 (Figure 9C). Further pH stability analysis showed that the fusion enzyme retained 55% and 19% of its initial activity after incubation at pH 5.0 and pH 10.0 for 20 h, respectively (Figure 9D). These findings suggest that while the enzyme undergoes partial inactivation under both acidic and alkaline conditions, it exhibits considerably lower tolerance to alkaline environments. First-order decay fitting of the inactivation curves yielded half-lives (t_1/2_) of 24.2 ± 0.6 h at pH 5.0 and 8.3 ± 0.2 h at pH 10.0, with corresponding inactivation rate constants (k) of 0.0286 ± 0.0007 h^−1^ and 0.0835 ± 0.0013 h^−1^, respectively (Figure 9D).

### 3.8. Optimization of Critical Biotransformation Parameters

#### 3.8.1. Effect of Crude Enzyme Concentration

**Figure 10:**
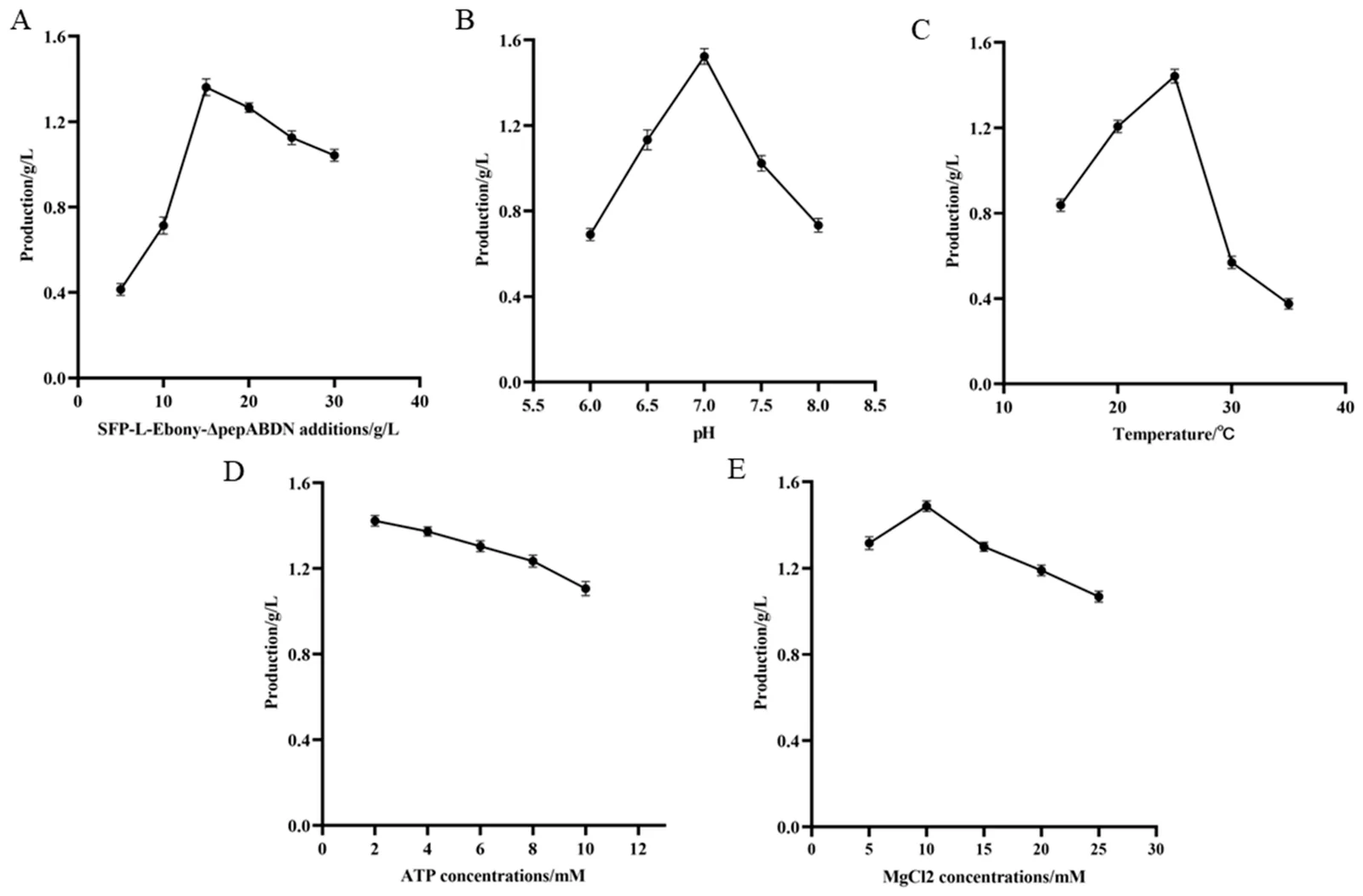
Optimization of biotransformation conditions. (**A**) Effect of SFP-L-Ebony-Δ*pepABDN*. (**B**) Effect of pH on biotransformation. (**C**) Effect of temperature on biotransformation. (**D**) Effect of ATP concentrations on biotransformation. (**E**) Effect of MgCl_2_ concentrations on biotransformation. Data are presented as mean ± SD, *n* = 3.

The experimental results showed that the maximum carcinine yield was achieved at a crude enzyme concentration of 15 g/L (Figure 10A). This suggests that crude enzyme concentration below 15 g/L was insufficient to meet the demands of efficient substrate conversion. Conversely, crude enzyme concentrations exceeding 15 g/L led to a decline in yield, which may be attributed to macromolecular crowding effects and non-specific binding of substrates or cofactors by excess crude enzyme proteins, thereby reducing their effective concentrations for the enzymatic reaction [41,42].

#### 3.8.2. Effect of pH

Fluctuations in pH can significantly alter the ionization states of both enzyme and substrate molecules, consequently affecting substrate binding and catalytic efficiency [43,44]. Moreover, extreme pH conditions can compromise enzyme stability, inducing conformational changes or even complete denaturation, ultimately leading to the loss of enzymatic activity [45]. To maximize biotransformation efficiency, reactions were systematically investigated across a pH range of 6.0–8.0. The results showed that under slightly acidic conditions, the carcinine yield remained low (Figure 10B), suggesting that the catalytic activity of the fusion enzyme SFP-L-Ebony-Δ*pepABDN* was inhibited in acidic environments. As the pH increased, the carcinine yield rose progressively, peaking at pH 7.0, after which a decline was observed. These findings indicate that the optimum biotransformation pH is 7.0, reflecting a preference of the enzyme system for neutral environments and underscoring the necessity of stringent pH control during the biotransformation process.

#### 3.8.3. Effect of Temperature

Temperature governs both enzyme kinetic reaction rates and thermodynamic stability; within a certain threshold, reaction rates accelerate with rising temperature, whereas excessive heat compromises structural integrity [46]. In this study, biotransformation was evaluated across a temperature range of 15–35 °C. The experimental results showed that the highest carcinine yield was obtained at 25 °C (Figure 10C), indicating that the fusion enzyme SFP-L-Ebony-Δ*pepABDN* is highly thermosensitive. When the temperature was elevated to 30 °C, the catalytic activity of the fusion enzyme decreased substantially, leading to a marked reduction in substrate utilization and diminished capacity for carcinine biosynthesis.

#### 3.8.4. Effect of ATP Concentration

In this multi-enzyme system, ATP is strictly required as a cosubstrate to activate β-alanine and is cleaved to AMP and PPi during the condensation reaction. Evaluation of different ATP concentrations showed that 2 mM ATP was the optimal concentration, yielding the highest carcinine titer (Figure 10D), which we attribute to endogenous ATP regeneration in the crude enzyme system. The crude lysate retains endogenous high-energy phosphate donors and a complete recycling enzyme network. Ebony cleaves ATP to AMP and PPi, after which endogenous PPase rapidly hydrolyzes PPi to inorganic phosphate, driving the condensation reaction strongly forward. Meanwhile, Adk converts AMP to ADP via phosphotransfer, and the resulting ADP is rephosphorylated to ATP through residual glycolytic flux and host kinases utilizing endogenous high-energy reserves such as PEP and polyphosphate. This cooperative network sustains continuous ATP recycling, effectively amplifying the utility of the exogenous ATP through multiple rounds of adenylate cycling.

#### 3.8.5. Effect of MgCl_2_ Concentration

Mg^2+^ typically participates in the adenylation process as a Mg-ATP complex and concurrently serves as an essential activator for SFP, thus fulfilling a dual role in this cascade system [47]. Biotransformation was conducted within an MgCl_2_ concentration range of 5–25 mM. As the Mg^2+^ concentration increased from 5.0 mM to 10.0 mM, the final yield of carcinine was enhanced accordingly (Figure 10E). This enhancement is likely because the elevated Mg^2+^ levels ensured an adequate supply of ions to activate the SFP enzyme while simultaneously complexing with ATP to form the active Mg-ATP state that drives the adenylation step. However, when the Mg^2+^ concentration exceeded 10.0 mM, the production yield began to decline.

### 3.9. Analysis of Scale-Up Biotransformation

**Figure 11:**
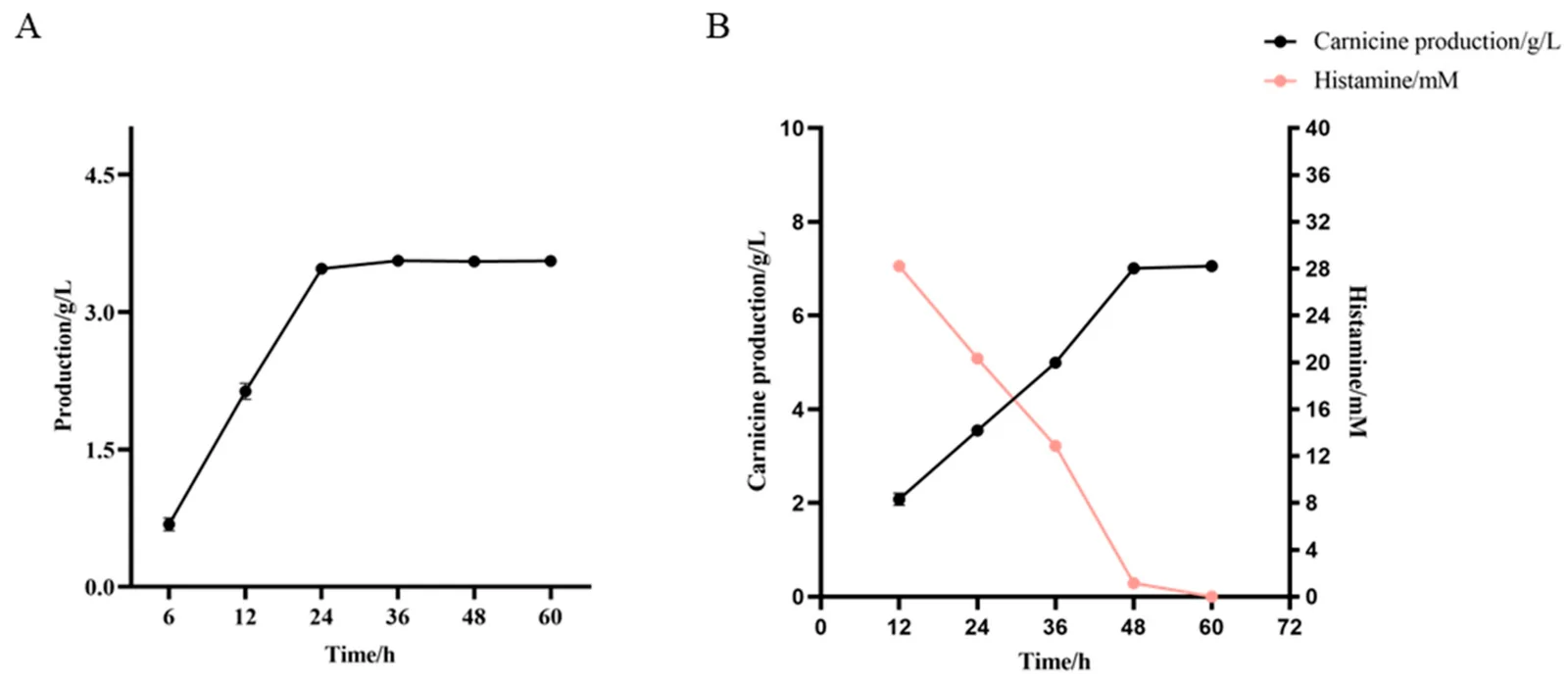
Expanding the biological transformation reactions of the system. (**A**) Biotransformation for the synthesis of carcinine under optimal reaction conditions. (**B**) Fed-batch biotransformation for the synthesis of carcinine under optimal reaction conditions. Data are presented as mean ± SD, *n* = 3.

To evaluate the industrial feasibility of the developed process, the biotransformation was scaled up to a 50-mL reaction volume with elevated initial substrate concentrations of 20 mM β-alanine and 40 mM histamine, using the engineered protease-deficient strain SFP-L-Ebony-Δ*pepABDN* under optimized conditions. Under this batch setup, the substrates were completely converted into carcinine within 24 h, achieving a titer of 3.47 g/L (Figure 11A). To further enhance substrate utilization and cumulative product titer, a fed-batch strategy was employed by adding 20 mM β-alanine and 2 mM ATP at the 24 h mark. With this supplement, the biotransformation proceeded efficiently, and the substrates were completely consumed within 48 h, yielding a final carcinine titer of 7.0 g/L (Figure 11B). The titer obtained in this work is comparable to the 7.34 g/L reported by Zhao et al. [23], yet remains below the 71.13 mM (approximately 13.0 g/L) achieved through recent linker engineering optimization [25].

## 4. Discussion

In this study, an efficient biosynthetic system for carcinine was constructed through the integrated optimization of a fusion protein strategy, host protease deficiency engineering, and a fed-batch fermentation process. Ebony existed almost entirely as inclusion bodies under conventional induction conditions, and neither reducing induction temperature nor IPTG concentration significantly ameliorated this phenomenon, suggesting that its folding impediment primarily arose from its intrinsic sequence characteristics. Upon fusion of the retro-protein XXA tag to the C-terminus, Ebony achieved efficient soluble expression at 30 °C while retaining its catalytic activity. This result is consistent with the mechanism proposed by Xie et al. [26], in which the XXA tag promotes correct folding by forming an antiparallel coiled-coil structure that shields exposed hydrophobic surfaces. Compared with conventional solubility-enhancing tags, XXA is characterized by lower molecular weight, higher solubilizing efficacy, and minimal interference with the native conformation of the fusion partner, thereby offering a more broadly applicable solution for the expression of recalcitrant NRPS enzymes.

In two-enzyme cascade reactions, the spatial arrangement of enzymes directly influences catalytic efficiency. In this study, a flexible linker (GGGGSGGGS) was employed to fuse SFP-3 and Ebony-C in tandem, covalently tethering the activating enzyme and the synthetase within a single polypeptide chain. This strategy increased the product titer by 1.44-fold compared with the two-enzyme independent expression systems. Recent studies have demonstrated that rational design of the linker peptide connecting SFP and Ebony (e.g., D5, L2, and L3 linkers) can enhance conversion efficiency by up to 3.5-fold relative to the conventional WSGE strain [25], underscoring the critical importance of fine-tuning the linker sequence for optimal fusion enzyme activity.

Furthermore, the peptidases encoded by *pepA*, *pepB*, *pepD*, and *pepN* in *E. coli* constitute the primary enzymatic system responsible for the degradation of exogenous dipeptides. In this study, a quadruple gene-deletion strain was constructed using CRISPR/Cas9 technology, which effectively suppressed the degradation pathway of carcinine without significantly affecting the expression level of the fusion protein. Jing et al. reported that knockout of *pepA*, *pepD*, and *pepN* using CRISPR/Cas9 increased the accumulation of Ala-Gln to 129% of that in the control strain [30], further corroborating the promoting effect of eliminating endogenous peptidase interference on dipeptide accumulation.

Enzymatic characterization showed that the optimal reaction temperature and pH for the fusion enzyme SFP-L-Ebony-Δ*pepABDN* were 25 °C and 7.0, respectively, consistent with the findings of Zhao et al. [23]. In a 50 mL system employing a fed-batch strategy, the carcinine titer reached 7.0 g/L, representing an approximately 2-fold increase compared with single-batch operation. Compared with analogous studies in the literature, the titer obtained in this study is comparable to the 7.34 g/L reported by Zhao et al., yet remains below the 71.13 mM (approximately 13.0 g/L) achieved through recent linker engineering optimization [25].

Compared with previous studies, our integrated approach offers several advantages, including the use of a crude enzyme system that simplifies preparation, the XXA tag that enables soluble expression of Ebony, and systematic peptidase knockout that minimizes product degradation. However, several limitations of the current system should be acknowledged. First, although the XXA tag significantly enhanced the soluble expression of Ebony, partial aggregation into inclusion bodies remained unavoidable under the tested conditions, which may reduce the yield of functional enzyme. Second, while the fed-batch strategy proved effective at the 50 mL scale, further process engineering is required for larger bioreactor operations. Future work should focus on enzyme immobilization to improve operational stability and reusability, which would also facilitate continuous processing and reduce overall production costs. Addressing these constraints will guide future optimization of the system.

In summary, this study successfully established an efficient biosynthetic system for carcinine through the integrated optimization of a fusion protein strategy, host protease deficiency engineering, and a fed-batch fermentation process. It systematically validated the feasibility of the XXA tag in promoting soluble expression of Ebony, the spatial synergy of the flexible linker-mediated dual-enzyme tethering, and the beneficial effect of endogenous peptidase knockout on dipeptide accumulation. Nevertheless, the current titer of 7.0 g/L remains below the economic threshold for industrial production, and issues concerning mass transfer, oxygen supply, and pH maintenance during scale-up cultivation warrant systematic investigation. Future research may be directed toward linker engineering optimization, metabolic flux analysis, and fermentation process scale-up, with the aim of advancing the industrialization of carcinine biomanufacturing.

## 5. Conclusions

In this study, a multi-enzyme cascade catalytic system comprising Ebony and SFP-3 was developed for the green biosynthesis of carcinine. By fusing a retro-protein XXA tag to the C-terminus of Ebony, the persistent issue of inclusion body formation during heterologous expression in *E. coli* was effectively overcome. A flexible linker was employed to spatially fuse SFP-3 and Ebony, thereby achieving functional synergy between the two enzymes and resulting in a 1.44-fold increase in product yield compared with the two-enzyme independent expression system. Through systematic CRISPR/Cas9-mediated knockout of four endogenous peptidase genes (*pepA*, *pepB*, *pepD*, and *pepN*), a quadruple protease-deficient strain was generated, which effectively inhibited product degradation. Under optimized conditions (crude enzyme concentration 15 g/L, ATP 2 mM, MgCl_2_ 10 mM, pH 7.0, 25 °C) and a fed-batch feeding strategy, the carcinine titer reached 7.0 g/L within 48 h. The strategies developed in this study, including XXA tag-mediated solubility enhancement, flexible linker-based enzyme fusion, and CRISPR/Cas9-guided peptidase deletion, provide a broadly applicable engineering framework for the sustainable production of other bioactive dipeptides in pharmaceutical, cosmeceutical, and functional food industries. This integrated engineering paradigm, encompassing fusion protein expression, host protease deficiency engineering, and fed-batch optimization, provides a feasible technological pathway and a valuable engineering framework for the industrial biosynthesis of carcinine and other high-value non-ribosomal peptide derivatives.

## Data Availability

The original contributions presented in this study are included in the article. Further inquiries can be directed to the corresponding author.

## Supplementary Materials

The following supporting information can be downloaded at: https://www.mdpi.com/article/10.3390/biom16081124/s1, Figure S1: Colony PCR analysis of strains by gel electrophoresis; Figure S2: HPLC profiles of carcinine and histamine; Figure S3: Calibration curve for carcinine dihydrochloride quantification by HPLC; Figure S4 Uncropped SDS-PAGE gels corresponding to Figure 4, Figure 5 and Figure 6, and Figure 8 in the main text; Table S1: Bacterial strains used in this study; Table S2: Primers used in this study for Crispr/Cas9.
