# Supplementary material for: Construction of Engineered Escherichia coli and Optimization of Conditions for Carcinine Synthesis via Multi-Enzyme Cascade Catalysis
Source: Biomolecules. 2026 Aug 1;16(8):1124. doi: 10.3390/biom16081124 (PMC13510768; doi:10.3390/biom16081124)
Supplement: Supplementary file 1 [file biomolecules-16-01124-s001.zip › biomolecules-4428002-supplementary.pdf]

Construction of Engineered *Escherichia coli* and Optimization of Conditions for Carcinine Synthesis via Multi-Enzyme Cascade Catalysis

Haoni Luan[a,b,c], Rui Yang[a,b,c], Wenhan Qiu[a,b,c], Kaiyue Feng[d], Wei Xu[a,b,c], Fei Wang[a,b,c], Wei Feng[a,b,c], Peng Song[a,b,c,*]

a School of Pharmaceutical Sciences and Food Engineering, Liaocheng University, Liaocheng 252000, China

b Shandong Key Laboratory of Applied Technology for Protein and Peptide Drugs, Liaocheng 252000, China

c State Key Laboratory of Macromolecular Drugs and Large-scale Preparation, Liaocheng 252000, China

d College of Agriculture and Biology, Liaocheng University, Liaocheng 252000, China

* Corresponding author: Peng Song, School of Pharmaceutical Sciences and Food Engineering, Liaocheng University, Liaocheng, China, Email: songpeng@lcu.edu.cn

Table S1. Bacterial strains used in this study

Strains	Detail	Source
BL21(DE3)	*E.coli* BL21(DE3)	Our lab
BL21-Ebony	*E.coli* BL21(DE3) pEt28a(+)-Ebony	This work
BL21-SFP-1	*E.coli* BL21(DE3) pEt28a(+)-SFP-1	This work
BL21-SFP-2	*E.coli* BL21(DE3) pEt28a(+)-SFP-2	This work
BL21-SFP-3	*E.coli* BL21(DE3) pEt28a(+)-SFP-3	This work
BL21-Ebony-C	*E.coli* BL21(DE3) pEt28a(+)-Ebony-C-His	This work
BL21-Ebony-N	*E.coli* BL21(DE3) pEt28a(+)-Ebony-N-His	This work
BL21(DE3)-SFP-L-Ebony	*E.coli* BL21(DE3) pEt28a(+)-SFP-3-GGGGSGGGS-Ebony-C	This work
BL21(DE3)-*pepB*	*E.coli* BL21(DE3)-Δ*pepB*	This work
BL21(DE3)-*pepBD*	*E.coli* BL21(DE3)-Δ*pepB* Δ*pepD*	This work
BL21(DE3)-*pepBDA*	*E.coli* BL21(DE3)-Δ*pepB* Δ*pepD* Δ*pepA*	This work
BL21(DE3)-*pepABDN*	*E.coli* BL21(DE3)-Δ*pepA* Δ*pepB* Δ*pepD* Δ*pepN*	This work
SFP-L-Ebony-Δ*pepABDN*	*E.coli* BL21(DE3)-SFP-3-L-Ebony-Δ*pepA* Δ*pepB* Δ*pepD* Δ*pepN*	This work

Table S2. Primers used in this study for Crispr/Cas9

Name	Type	Primer(5'→3')
*pepA*1	Primer for donor DNA construction and validation (*pepA*)	TTACTCTTCGCCGTTAAACCCAGC
*pepA*2	Primer for donor DNA construction and validation (*pepA*)	ATGGAGTTTAGTGTAAAAAGCGGTAGCC
*pepB*1	Primer for donor DNA construction and validation (*pepB*)	ATGACAGAAGCGATGAAAATTACCCTC
*pepB*2	Primer for donor DNA construction and validation (*pepB*)	TTACGCCGTTAACAGATTAGCTATCGTG
*pepD*1	Primer for donor DNA construction and validation (*pepD*)	GTGTCTGAACTGTCTCAATTATCTCCACAG
*pepD*2	Primer for donor DNA construction and validation (*pepD*)	TTACTTCGCC GGAATTTCTTTCAG
*PepN*1	Primer for donor DNA construction and validation	ATGACTCAACAGCCACAAGCCAAATAC

	(*pepN*)	
*pepN*2	Primer for donor DNA construction and validation (*pepN*)	TCAAGCCAGTGCTTTAGTTATCTTCTCGTAC
*pepA*F	sgRNA for *pepA*	TAGTACACGTCGCACAGTACCAGG
*pepA*R	sgRNA for *pepA*	AAACCCTGGTACTG TGCGACGTGT
*pepB*F	sgRNA for *pepB*	TAGTTGCGGCGACCCTCACCGGGG
*pepB*R	sgRNA for *pepB*	AAACCCCCGGTGAG GGTCGCCGCA
*pepD*F	sgRNA for *pepD*	TAGTCGCCTTATCGATTTCAACGG
*pepD*R	sgRNA for *pepD*	AAACCCGTTGAAAT CGATAAGGCG
*pepN*F	sgRNA for *pepN*	TAGTGCTGGCGGCGCTTTCTGCGG
*pepN*R	sgRNA for *pepN*	AAACCCGCAGAAAG CGCCGCCAGC

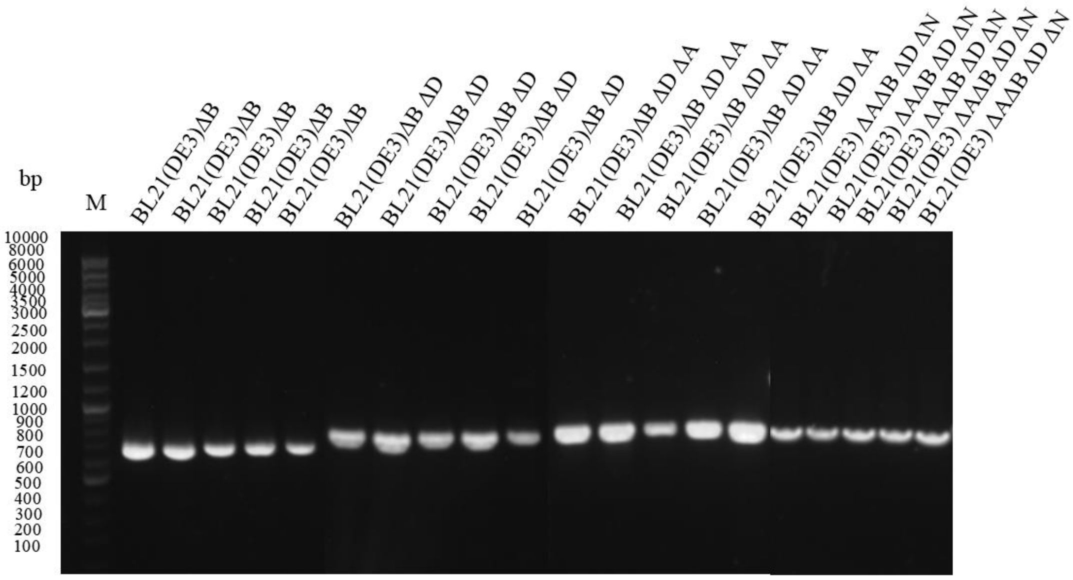

Figure S1 Colony PCR analysis of strains by gel electrophoresis.

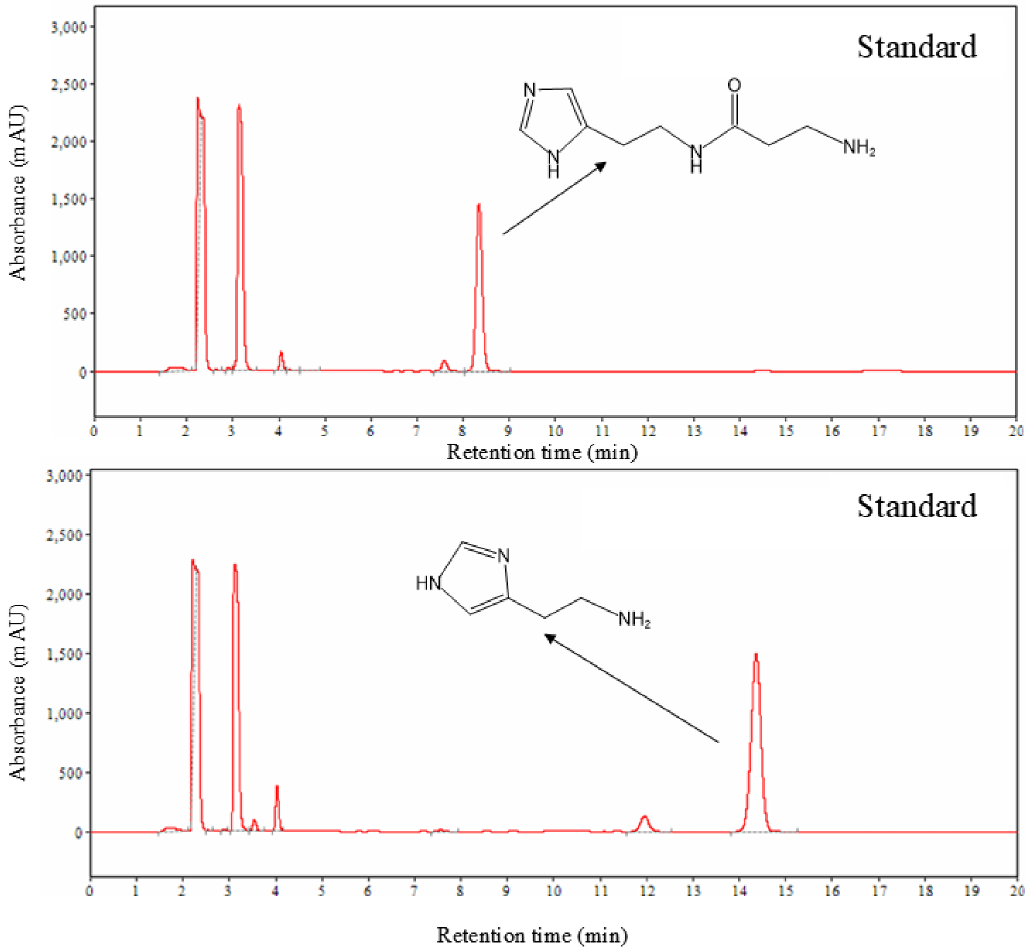

Figure S2 HPLC profiles of carcinine and histamine.

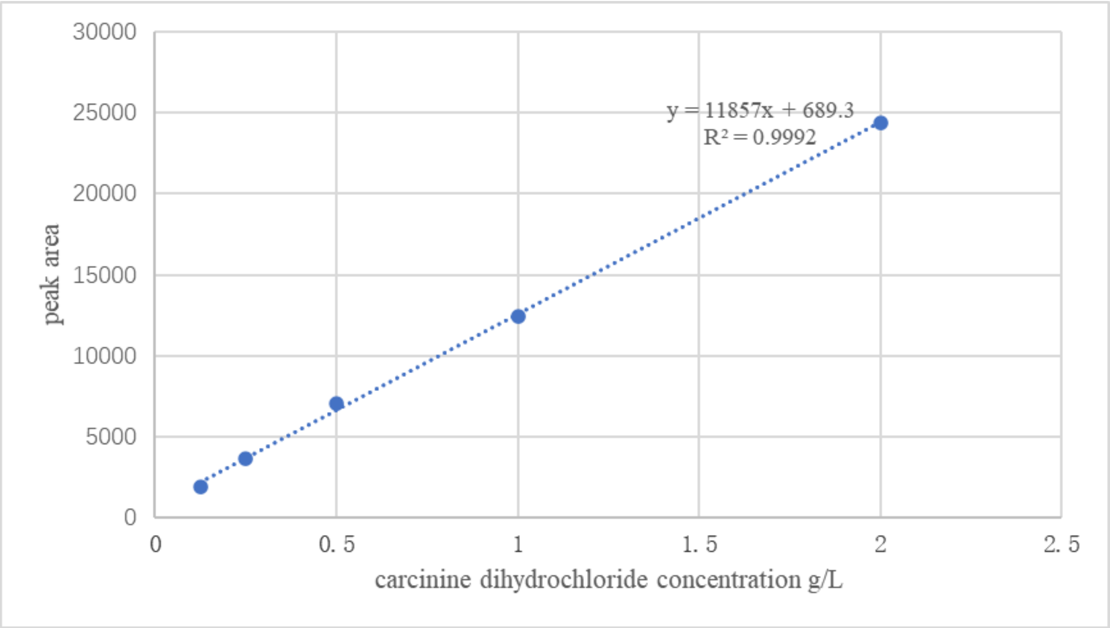

Figure S3 Calibration curve for carcinine dihydrochloride quantification by HPLC

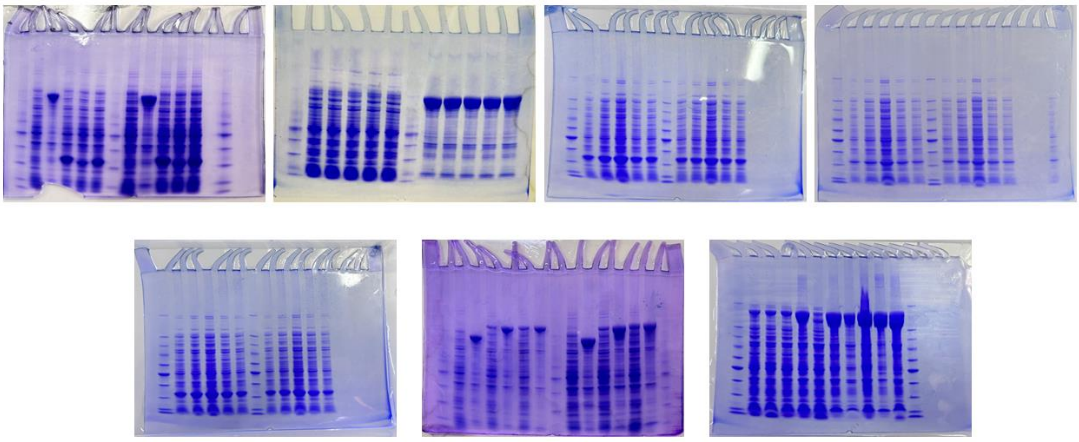

Figure S4 Uncropped SDS-PAGE gels corresponding to Figures 4, 5, 6, and 8 in the main text
